# Digital interventions for genomics and genetics education, empowerment, and service engagement: A systematic review

**DOI:** 10.1007/s12687-023-00648-w

**Published:** 2023-05-18

**Authors:** Norina Gasteiger, Amy Vercell, Naz Khan, Dawn Dowding, Angela C. Davies, Alan Davies

**Affiliations:** 1 Division of Nursing, Midwifery and Social Work, School of Health Sciences, The University of Manchester, Manchester, UK; 2 Division of Informatics, Imaging and Data Sciences, School of Health Sciences, The University of Manchester, Manchester, UK; 3 The Christie NHS Foundation Trust, Wilmslow Road, Manchester, M20 4BX UK; 4 Genomic Medicine, St. Mary’s Hospital, Manchester Academic Health Science Centre, Central Manchester University Hospitals NHS Foundation Trust, Manchester, M13 9WL UK

**Keywords:** Genetic counselling, Genetics education, Genetic services, Human genetics, Digital technology, Digital health

## Abstract

**Background:**

Patient-facing digital technologies may reduce barriers to and alleviate the burden on genetics services. However, no work has synthesised the evidence for patient-facing digital interventions for genomics/genetics education and empowerment, or to facilitate service engagement more broadly. It is also unclear which groups have been engaged by digital interventions.

**Aim:**

This systematic review explores which existing patient-facing digital technologies have been used for genomics/genetics education and empowerment, or to facilitate service engagement, and for whom and for which purposes the interventions have been developed.

**Methods:**

The review adhered to the Preferred Reporting Items for Systematic reviews and Meta-Analyses guidelines. Eight databases were searched for literature. Information was extracted into an Excel sheet and analysed in a narrative manner. Quality assessments were conducted using the Mixed Methods Appraisal Tool.

**Results:**

Twenty-four studies were included, of which 21 were moderate or high quality. The majority (88%) were conducted in the United States of America or within a clinical setting (79%). More than half (63%) of the interventions were web-based tools, and almost all focussed on educating users (92%). There were promising results regarding educating patients and their families and facilitating engagement with genetics services. Few of the studies focussed on empowering patients or were community-based.

**Conclusion:**

Digital interventions may be used to deliver information about genetics concepts and conditions, and positively impact service engagement. However, there is insufficient evidence related to empowering patients and engaging underserved communities or consanguineous couples. Future work should focus on co-developing content with end users and incorporating interactive features.

**Supplementary Information:**

The online version contains supplementary material available at 10.1007/s12687-023-00648-w.

## Introduction

It has been estimated that 5.3% of new-borns will have genetic disorders (Verma & Puri 2015), with 30,000 children in the United Kingdom (UK) alone receiving a diagnosis every year (Gene People 2020; Genetic Alliance 2021). These can occur through genetic variants, which are heritable changes to the DNA sequence and may involve one or multiple genes (Richards et al. 2015). However, genetic disorders do not affect all populations equally. Research has shown a greater prevalence of genetic disorders in developing countries and non-Western populations due to higher numbers of consanguineous marriages (Posch et al. 2012; Verma & Puri 2015). This pattern is also observed in developed countries. For example, in the UK, the Born in Bradford cohort study with 12,453 women found that rates of consanguinity were significantly higher in Pakistani women, compared to their white British counterparts (37.5% vs. 0.0% for first-cousin marriage) (Bhopal et al. 2014). Genetic counselling with screening for genetic variants is essential to help identify possible genetic conditions, especially after marriage and before conception (Verma & Puri 2015). Additionally, given that some metabolic disorders require treatment with medication or lifestyle changes, newborn screening is essential. Improving understanding about genetic inheritance can empower affected families and reduce unexpected, affected births. However, significant unmet need for culturally sensitive genetic information and services leaves many families poorly supported.

General barriers to genetic counselling and related screening services have been reported in the literature. The stigmatisation of genetic disorders might deter individuals from accessing services due to potential discrimination, issues with seeking insurance, and blaming individuals and population groups (de Vries et al. 2020; Williams et al. 2010). Additionally, perceived religious and cultural barriers might affect engagement in communities where consanguineous marriages are practised (Alkuraya 2013). Genetic service-related barriers are also evident and were explored in a scoping review by Raspa et al. (2021). The scoping review identified key issues from the United States of America (USA) and abroad, including the limited number of genetic specialists, appointment waiting times, delivery by non-genetic providers, and reimbursements and obtaining a licence, concluding that health information technologies may help to overcome these barriers.

Digital technologies may help to educate, empower, and engage populations in genetic services, overcoming barriers related to stigmatisation and reducing the burden on genetic counselling services. Engagement can be patient-driven, whereby people may seek information online (Shepherd 2010) or on specific apps (Gasteiger et al. 2022; Talwar et al. 2019). Patients already send emails to genetics services, requesting information on things like access to testing and specialist advice regarding treatment (Shepherd 2010). Additionally, services may use technologies to distribute accurate and reliable information or to facilitate engagement with their services. For example, our systematic review of 22 patient-facing genetics/genomics apps uncovered that some apps allowed genetic testing kits to be purchased, with results and further information provided directly through the app (Gasteiger et al. 2022). There is also the possibility to provide genetic counselling via video teleconferencing (Gorrie et al. 2021) or over the phone (Tutty et al. 2019), and results could be provided through a patient portal linked to the electronic health record (Korngiebel et al. 2018).

While there is an abundance of literature exploring the use of technologies for genetics/genomics education and counselling, no work to date has synthesised the evidence for patient-facing digital interventions for genomics/genetics education and empowerment, or to facilitate service engagement more broadly. Related work has included a rapid systematic review to explore the benefits and disadvantages of telegenetics (genetic counselling via videoconferencing) (Gorrie et al. 2021) and a commentary on how digital tools can advance quality and equity in genomic medicine (Bombard & Hayeems 2020). We also conducted a systematic review of 22 UK patient-facing genetics/genomics apps, finding that there is a need for an accessible, culturally sensitive, and evidence-based app to improve genetic literacy within specific communities (Gasteiger et al. 2022). It is also unclear which groups and communities have been engaged by digital interventions and for which purposes.

### Research questions

Two research questions guided the review: 1) What existing patient-facing digital technologies have been used for genomics/genetics education and empowerment or to facilitate service engagement? 2) For whom (patient groups, populations, or communities) and for which purposes have the interventions been developed?

## Methods

We conducted a systematic review, which is reported in accordance with the updated Preferred Reporting Items for Systematic reviews and Meta-Analyses (PRISMA) 2020 guidelines (Page et al. 2021). The protocol was registered in the international prospective register of systematic reviews in August 2022 (PROSPERO: CRD42022348127).

### Search and screening process

#### Databases and keywords

Peer-reviewed literature was retrieved from PubMed, SCOPUS, Embase, Web of Science, CINAHL, PsycINFO, IEEE Xplore and the ACM Digital Library in June 2022. The supplementary file presents the specific search keywords and syntax for three database searches. Each keyword was separated by Boolean operators ‘AND’ and ‘OR.’ No limits were placed on the date of publication. However, where possible, limits were placed on the language (English) and species (human).

#### Inclusion and exclusion criteria

Qualitative, quantitative, and mixed-methods empirical studies published in English were included. The research had to report on a digital intervention for patients, families, the public, or communities used for genetics/genomics empowerment, education, or to facilitate service engagement.

Technical papers, non-empirical studies, theses, dissertations, conference proceedings, and grey literature were excluded. Research studies focussing on healthcare professionals (e.g., genetics counsellors, nurses, or doctors) were excluded unless information on patients or the public could be separated. Papers focussing on the development of interventions were also excluded, unless there was an evaluation component.

#### Screening and literature management

The Rayyan software (https://www.rayyan.ai/) was used to manage the literature. A two-step screening process was conducted, whereby two authors (NG and AV) first independently reviewed the abstract and titles of the literature for eligibility. The two authors then independently read the full-texts to determine eligibility. Disagreements on eligibility were resolved through discussion until a consensus was met. Duplicates were automatically detected and removed by Rayyan.

### Analysis

A data extraction sheet was developed in Microsoft Excel, into which relevant data were extracted (see Box 1). Descriptive statistics were generated where applicable, such as on the research design, participant sample (e.g., number of participants and countries), focus areas, and technologies used. We then conducted a formal narrative synthesis by broadly grouping and describing the studies by the following characteristics: 1) Target population characteristics; 2) intervention type, focus, and content; 3) Type of outcome (e.g., education, empowerment, service engagement).

Box 1. Information extracted from eligible research studies.

**Table:** 

• First author
• Year of publication
• Title of study
• Research design and methods
• Study objective
• Focus (genomics/genetics)
• Technology description
• Technology setting (e.g., part of service or independent)
• Setting; country
• Sample (type/population, size, age, gender, ethnicity)
• Outcome (education, knowledge, awareness, service engagement, understanding)
• Barriers/facilitators to use, adoption, uptake

### Quality appraisals

The evidence was appraised using the Mixed Methods Appraisal Tool (MMAT) (Hong et al. 2018). The MMAT can be used for qualitative, quantitative, and mixed-methods research. It consists of two screening questions and five study design-related questions, each of which is scored 1 (yes) or 0 (no). Similar to other reviews (Mogharbel et al. 2021; Pluye et al. 2009), we categorised each study as low quality (≤ 40%, i.e., met 1–2 criteria), moderate quality (60%-80%, i.e., met 3–4 criteria), or high quality (100%, i.e., met all 5 criteria). Each study was independently assessed by two authors (NG and AV). Interrater reliability was calculated using Cohen's kappa (κ) on IBM SPSS (version 27).

## Results

**Fig. 1:**
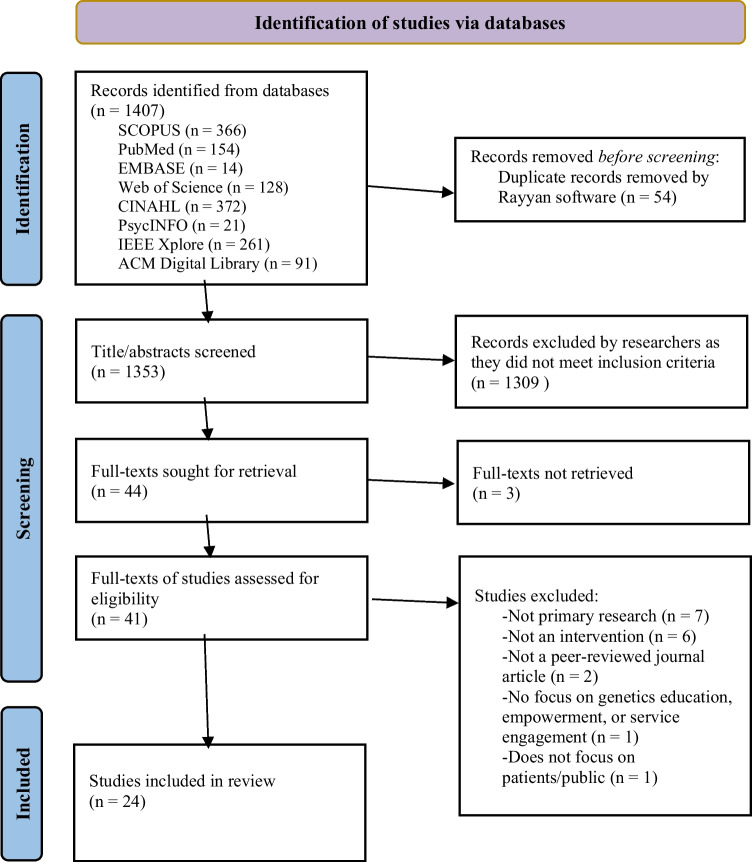
PRISMA flowchart showing the literature search and screening process

The database search yielded 1407 records, of which 54 duplicates were removed. Of the 1353 titles and abstracts screened, 1309 were excluded as they did not meet the inclusion criteria. Three of the 44 full-texts could not be retrieved, leaving 41 studies for full-text screening. Of these, 16 were not eligible due to the following reasons: not primary research (*n* = 7), not an intervention (*n* = 6), not a peer-reviewed journal article (*n* = 2), did not focus on genetics education, empowerment, or service engagement (*n* = 1), and does not focus on patients or the public (*n* = 1). Ultimately, 24 studies were included in the review. Figure 1 outlines the literature search and screening process.

### Characteristics and quality of the included studies

The 24 included research papers were published from 2008 (O'Neill et al. 2008) to 2022 (Bangash et al. 2022; Bradbury et al. 2022; Christian et al. 2022). Fifteen of the studies were experimental, and nine were observational. Specifically, 11 were randomised controlled trials (Adam et al. 2018; Biesecker et al. 2018; Bowen et al. 2011; Bradbury et al. 2022; Cragun et al. 2020; Gornick et al. 2018; Hernan et al. 2020; Prado et al. 2018; Vogel et al. 2019; Wang et al. 2021; Wierstra et al. 2018), six were mixed methods studies (Bangash et al. 2022; Boudreault et al. 2017; Brown-Johnson et al. 2021; Schmidlen et al. 2019; Solomon et al. 2020; Williams et al. 2018), four were non-randomised quantitative studies (Christian et al. 2022; Conijn et al. 2020; Hardy et al. 2018; O'Neill et al. 2008), two were descriptive quantitative studies (Beaudoin et al. 2011; Nazareth et al. 2021) and one was qualitative (Suckiel et al. 2021). Table 1 and the supplementary file provide more information on the study designs, including the sample and data collection methods.

**Table 1:** Summary of the included studies

First author; date	Setting; country	Sample; sample size	Study design; data collection methods	Technology	Quality
Adam et al. 2018	Tertiary care children’s hospital; Canada	Parents of children with early‐onset epilepsy of unknown cause; 106	Randomised controlled trial (RCT); surveys	Web-based tool (DECIDE)	Moderate
Bangash et al. 2022	Zoom/online; public access or via Mayo Clinic; USA	Patients (probands with familial hypercholesterolemia diagnosis); 9	Mixed methods; talk aloud; surveys	Web-based tool (FH Family Share)	Moderate
Beaudoin et al. 2011	University of Utah Metabolic Service Clinic; USA	Adult patients and parents/guardians of newborns/children with metabolic conditions; 53	Quantitative (descriptive); surveys	Online resource, information prescription (Genetics Home Reference)	Moderate
Biesecker et al. 2018	Clinical (National Institutes of Health); USA	Adults (recruited from the ClinSeq cohort- part of a clinical research cohort for exome sequencing); 459	RCT; surveys	Web-based platform	Moderate
Boudreault et al. 2017	Deaf community, online, conference; USA	ASL speakers; Focus groups: 43; questionnaires: 97	Mixed methods; focus groups; questionnaire	Cancer Genetics Education Module video	Moderate
Bowen et al. 2011	Community, online; USA	Community sample (women); 1354	RCT; surveys	Website	High
Bradbury et al. 2022	Community and academic practices; USA	Patients with advanced cancer undergoing tumour only sequencing; 472	RCT; surveys	Web-based tool (Communication and Education in Tumour Profiling)	Moderate
Brown-Johnson et al. 2021	Academic primary care clinic in community; USA	Patients; 50 (16 patients took part in interviews)	Mixed methods; chart review; interviews	Multi-modal precision health platform	Moderate
Christian et al. 2022	2 tertiary and 3 outreach sites; Canada	Patients with a clear/suspected diagnosis of hypertrophic cardiomyopathy; 82	Non-randomised controlled trial; surveys	Webinar	Moderate
Conijn et al. 2020	Public, online; Netherlands	Participants in the reproductive age, 18–45 years; 1570	Non-randomised, historically controlled study; surveys	Educational video	High
Cragun et al. 2020	Vanderbilt Hereditary Cancer Clinic; USA	Patients with a personal and/or family history of various cancers; 305	RCT; surveys	Web-based tool	Moderate
Gornick et al. 2018	22 surgical practices in 4 states; USA	People with early early-stage breast cancer; 537	RCT; surveys	Decision tool (iCan-Decide)	High
Hardy et al. 2018	10 universities; USA	Undergraduate Jewish students; 1794	Quantitative non-randomized (pre posttest); surveys	Video and website (JScreen)	Low
Hernan et al. 2020	Division of Clinical Genetics at the Children’s Hospital of NYC; USA	Parents of patients being offered clinical exome sequencing (CES); 207	RCT; surveys	Video	Moderate
Nazareth et al. 2021	180 clinics; USA	Patients scheduled for appointments at 180 clinics; 61,070	Quantitative descriptive (observational); surveys, use metrics	Chatbot (Genetic Information Assistant- GIA)	Moderate
O'Neill et al. 2008	Thoracic Oncology Clinic at the H. Lee Moffitt Cancer Center and Research Institute; USA	Patients and relative-smokers; 232 (116 patients, 116 relative-smokers)	Quantitative non-randomised (cohort study); surveys	Web-based tool	Moderate
Prado et al. 2018	Large tertiary care medical centre; USA	First degree relatives of patients with rheumatoid arthritis; 238	RCT; surveys	Web-based tool (Personalized Risk Estimator for RA)	Moderate
Schmidlen et al. 2019	Community; USA	Community/public—general genetic health; 62	Mixed methods; focus groups, survey	Chatbots (consent, follow-up &, cascade chatbot)	Low
Solomon et al. 2020	City of Hope National Medical Center; USA	Patients with cancer; 13 (8 patients, 5 family members)	Mixed methods; focus groups, survey	Web-based tool	Moderate
Suckiel et al. 2021	Clinical settings; USA	Paediatric neurologic, immunologic, or cardiac disorders; 18	Qualitative; interviews	Application (Genomic Understanding, Information and Awareness- GUÍA)	Moderate
Vogel et al. 2019	Gynecologic Cancer Clinic; USA	Women (untested with a history of epithelial ovarian, primary peritoneal or fallopian tube cancer); 104	RCT; surveys	Mobile application (mobile Application for Genetic Information on Cancer- mAGIC)	Moderate
Wang et al. 2021	Boston Medical Center; USA	Patients; 273	RCT; usage metrics, concordance between tool and genetic counsellor	Conversational agent (Virtual Counsellor for Knowing Your Family History- VICKY); web- based tool (My Family Health Portrait- MFHP)	High
Wierstra et al. 2018	Community, university and clinic; USA	Patients and people attending a clinic for inflammatory bowel disease; 78	RCT; surveys	Website	Low
Williams et al. 2018	Genetic counselling service; USA	Parents of children with undiagnosed Intellectual Disability, Autism Spectrum Disorder and/or multiple congenital anomalies; 52	Mixed methods; interviews; surveys	Application (GenomeCOMPASS report)	Moderate

### Quality appraisals

The MMAT (Hong et al. 2018) was used to assess the quality of the included studies. The MMAT considers five different study designs, with two pre-screening questions and five questions per study design used to assess quality. For example, this may consider whether randomisation was performed and whether groups are comparable at baseline for RCTs or whether components of mixed methods studies are effectively integrated to answer the research question.

There was almost perfect agreement between the two raters using the MMAT (95%; κ = 0.870 (95% CI, 0.768 to 0.972; *p* < 0.001). Of the 11 randomised controlled trials, seven were rated as moderate quality, three as high quality and one as low quality. Six did not blind outcome assessors to the intervention, and three did not have complete outcome data (≥ 80%). Five mixed methods studies were deemed as moderate in quality, and one of low quality. None of the mixed methods studies met all the criteria for qualitative and quantitative research, mostly due to lacking information on the analysis method for the quantitative data. Half of the non-randomised quantitative studies were considered moderate quality; one was considered low quality and one was considered high quality. Half did not have complete outcome data and did not appear to account for confounding variables (e.g., previous technology use, level of education and pre-existing knowledge of genetics). Both descriptive quantitative studies were considered of moderate quality as they were at-risk for nonresponse bias due to low response rates and significant differences between users and non-users of the digital intervention. The qualitative study was considered high quality.

### Target population characteristics

Of the 24 studies included, 21 (88%) were conducted in the USA, two (8%) were conducted in Canada, and one (4%) took place in the Netherlands (see Table 1). The sample sizes ranged from 9 to 61,070 participants (mean 2892, median 128). The recruitment settings were predominantly from within a clinical environment (*n* = 19, 79%), with one (4%) accessed either online or via a clinical setting (Bangash et al. 2022), three (13%) were available freely online (Conijn et al. 2020; Schmidlen et al. 2019; Wierstra et al. 2018) and one study recruited participants from within a university setting (4%) (Hardy et al. 2018).

The population focus for the studies varied. Those recruited from within a clinical setting tended to focus on a specific condition. Eight (33%) focussed on cancer (Bowen et al. 2011; Bradbury et al. 2022; Cragun et al. 2020; Gornick et al. 2018; Nazareth et al. 2021; O'Neill et al. 2008; Solomon et al. 2020; Vogel et al. 2019). Less than 10% focussed on exome sequencing (*n* = 2, 8%) (Biesecker et al. 2018; Hernan et al. 2020), metabolic conditions (*n* = 1, 4%) (Beaudoin et al. 2011), hypertrophic cardiomyopathy (*n* = 1, 4%) (Christian et al. 2022), rheumatoid arthritis (*n* = 1, 4%) (Prado et al. 2018), epilepsy (*n* = 1, 4%) (Adam et al. 2018), paediatric neurologic, immunologic, or cardiac disorders (*n* = 1, 4%) (Suckiel et al. 2021), autism (*n* = 1, 4%) (Williams et al. 2018), or irritable bowel disease (*n* = 1, 4%) (Wierstra et al. 2018).

One study (4%) had a broad focus on all health conditions (Brown-Johnson et al. 2021) and another (Wang et al. 2021) focused on underserved communities, looking at vulnerable patient populations, including racial/ethnic minorities. Studies which recruited the public online focussed on breast health (*n* = 1, 4%) (Bowen et al. 2011), expanded carrier screening and mucopolysaccharidosis type III (*n* = 1, 4%) (Conijn et al. 2020), American Sign Language (ASL) speakers in the deaf community (*n* = 1, 4%) (Boudreault et al. 2017), familial hypercholesterolemia (*n* = 1, 4%) (Bangash et al. 2022), and general genetic health and literacy (*n* = 1, 4%) (Schmidlen et al. 2019). One (4%) study focused on university students specifically focused upon the Jewish community (Hardy et al. 2018).

Demographic information was reported in some studies. Five (21%) of the studies were for women only, and for those that specified the gender of participants, male participation ranged from 2% (1242/61,070) to 67% (29/43). Age was not recorded in three of the studies. Ethnicity and race were not reported in six studies. In the studies that reported ethnicity or race, participation by white/Caucasian individuals ranged from 14.3% (39/273) to 100% (9/9). Only three studies had 50% or more of participants identifying as non-white (e.g., Hispanic/Latinx, African American or Asian). Characteristics of the included studies are further detailed in Table S3 in the supplementary file.

### Summary of the interventions

Of the 24 included papers, 15 (63%) interventions used a web-based tool. One also had a video component that included an animated educational video (Hardy et al. 2018). One was a multi-model precision health platform that used data-driven medicine to predict and prevent disease, with four associated components: health coaching, remote monitoring, pharmacogenomics, and genetic screening (Brown-Johnson et al. 2021). Three (13%) other interventions consisted of videos (Boudreault et al. 2017; Conijn et al. 2020; Hernan et al. 2020), three (13%) were mobile applications (apps) (Suckiel et al. 2021; Vogel et al. 2019; Williams et al. 2018), two (8%) were chatbots (Nazareth et al. 2021; Schmidlen et al. 2019), and one (4%) was a webinar (Christian et al. 2022). Most (*n* = 20, 83%) were used as part of a clinical service, which involved being recruited when attending for a planned consultation with a clinical team. This meant a patient may need to be referred by a clinician to access the platform, and/or the platform is used to enhance the clinical service, the goal being to assess the level of genetic literacy and the impact of the digital intervention compared to standard care. For the four (17%) platforms that are used independently from a clinical setting, these focussed on educating users on genetic health. Table S4 in the supplementary file provides a summary of the digital interventions.

Almost all the studies focussed on educating users through the digital interventions (*n* = 22, 92%) (Adam et al. 2018; Bangash et al. 2022; Beaudoin et al. 2011; Biesecker et al. 2018; Boudreault et al. 2017; Bowen et al. 2011; Bradbury et al. 2022; Christian et al. 2022; Conijn et al. 2020; Cragun et al. 2020; Gornick et al. 2018; Hardy et al. 2018; Hernan et al. 2020; Nazareth et al. 2021; O'Neill et al. 2008; Prado et al. 2018; Schmidlen et al. 2019; Solomon et al. 2020; Suckiel et al. 2021; Vogel et al. 2019; Wierstra et al. 2018; Williams et al. 2018). The majority (*n* = 17, 71%) also explored facilitating service engagement (Bangash et al. 2022; Beaudoin et al. 2011; Biesecker et al. 2018; Bowen et al. 2011; Brown-Johnson et al. 2021; Christian et al. 2022; Conijn et al. 2020; Cragun et al. 2020; Hardy et al. 2018; Nazareth et al. 2021; O'Neill et al. 2008; Schmidlen et al. 2019; Solomon et al. 2020; Suckiel et al. 2021; Vogel et al. 2019; Wang et al. 2021; Williams et al. 2018). Only two (8%) intended to empower users (Adam et al. 2018; Cragun et al. 2020). The specific impacts on outcomes are reported later in the paper. Table 2 summarises the characteristics of the interventions.

**Table 2:** Characteristics of the interventions from the reviewed literature (*N* = 24)

Characteristic	Number (%)
Intervention type	
Web-based tool	15 (63)
Video	3 (13)
Mobile application	3 (13)
Chatbot	2 (8)
Webinar	1 (4)
Integration with service	
Part of clinical service	20 (83)
Not part of clinical service	4 (17)
Intervention focus	
Educational	22 (92)
Facilitate service engagement	17 (71)
Empowerment	2 (8)

#### Barriers and facilitators to uptake

Some authors acknowledged potential barriers to uptake and explored differences in demographics between users and non-users. Users tended to be younger (O'Neill et al. 2008), more motivated to change health behaviours (O'Neill et al. 2008) and have a higher level of health literacy (Wang et al. 2021). Barriers included not being able to use a computer (Wang et al. 2021), having a lower grade reading age than grade 12 (Wierstra et al. 2018) and being confused as to how to use the tool (Brown-Johnson et al. 2021; Wang et al. 2021; Williams et al. 2018). Strategies to increase participation included providing equipment (e.g., devices, genetic tests, associated counselling) at no cost to participants (Brown-Johnson et al. 2021) or providing a free meal (Hardy et al. 2018).

### Outcomes of the interventions

#### Education, knowledge and understanding

A majority (*n* = 19, 79%) of the studies reported on changes to knowledge or understanding of genetic concepts (Adam et al. 2018; Beaudoin et al. 2011; Biesecker et al. 2018; Boudreault et al. 2017; Bowen et al. 2011; Bradbury et al. 2022; Christian et al. 2022; Conijn et al. 2020; Cragun et al. 2020; Gornick et al. 2018; Hardy et al. 2018; Hernan et al. 2020; O'Neill et al. 2008; Prado et al. 2018; Schmidlen et al. 2019; Solomon et al. 2020; Vogel et al. 2019; Wierstra et al. 2018; Williams et al. 2018). Three other studies did not directly report on changes to knowledge or understanding but instead focussed on experiences with the educational content (Bangash et al. 2022; Nazareth et al. 2021; Suckiel et al. 2021). For example, one stated that 71.4% of 61,070 users completed the genetic testing education section of the clinical chatbot and reported high acceptability with the content (Nazareth et al. 2021). In the study by Bangash et al. (2022) 56% (5/9) of the participants found the information easy to find and 78% (7/9) found it very easy to understand. Responses to the intervention in Suckiel et al. (2021) were also positive, with Spanish-speaking participants valuing the opportunity to read the content in their preferred language.

Sixteen studies reported positive effects on knowledge, whereby the understanding of genetics information (e.g., genetic testing, genetic health conditions, recessive inheritance) improved due to the digital intervention (Adam et al. 2018; Beaudoin et al. 2011; Biesecker et al. 2018; Bowen et al. 2011; Bradbury et al. 2022; Christian et al. 2022; Conijn et al. 2020; Cragun et al. 2020; Gornick et al. 2018; Hardy et al. 2018; Prado et al. 2018; Schmidlen et al. 2019; Solomon et al. 2020; Vogel et al. 2019; Wierstra et al. 2018; Williams et al. 2018). This was measured through knowledge quizzes or self-reported changes by participants. For example, in one qualitative study, participants remarked that they could take their time with the chatbot, which helped them understand the information. Many participants also stated that they learned more using the chatbot than when speaking to a person in the clinic. Some studies also found the digital interventions equivalent to, or noninferior to genetic counsellors regarding knowledge outcomes. Biesecker et al. (2018) reported their web-based platform as noninferior immediately after the intervention, one month later and six months later. Adam et al. (2018) also noted that participants in the DECIDE intervention group and genetic counselling control groups had increased knowledge, with DECIDE appearing equivalent at conveying information.

Three studies specifically mentioned that knowledge could be shared with others, including family and friends. Boudreault et al. (2017) reported that participants were motivated to tell their family and friends about the educational video, while participants in the study by Solomon et al. (2020) wanted to share information with their family, friends and other patients with cancer. Lastly, participants thought the cascade chatbot in the study by Schmidlen et al. (2019) could help share genomic test results and answer their relative's questions as it knows “all of the relevant clinical details regarding the results.” This could be achieved through the Family Sharing Tool which deploys a link to share genetic test results with others via text, email or Facebook Messenger.

A few studies reported unexpected negative findings, whereby there were no significant differences in knowledge outcomes between those who did and those who did not use the digital intervention (Hernan et al. 2020; O'Neill et al. 2008). In one study, knowledge scores were significantly lower in parents in the intervention group who watched an educational video before a genetic counselling appointment, compared to the control group who did not watch the video (Hernan et al. 2020). Boudreault et al. (2017) also found that participants in their study did not fully comprehend the material conveyed, with participants highlighting the need for the messaging to be improved by reducing the amount of content and using simpler language.

Participants across other studies also suggested improving the content within the digital interventions. Recommendations included adding more information about how to respond to health problems experienced by children with specific disorders, genetic screening for children, treatment options, risk of disease, testing options for family, the frequency and prevalence of the variant, and an explanation for why testing negative would not remove all future disease risk (Bangash et al. 2022; Beaudoin et al. 2011; Biesecker et al. 2018; Schmidlen et al. 2019). Participants in one study also disagreed on the framing of the content, whereby some believed prognostic information should convey a sense of hope, while others desired more factual information (Solomon et al. 2020).

#### Service engagement

Seventeen studies (71%) reported on direct or expected impacts to service engagement or the delivery of clinical genetics healthcare (e.g., testing, diagnosis, and counselling) (Bangash et al. 2022; Beaudoin et al. 2011; Biesecker et al. 2018; Bowen et al. 2011; Brown-Johnson et al. 2021; Christian et al. 2022; Conijn et al. 2020; Cragun et al. 2020; Hardy et al. 2018; Nazareth et al. 2021; O'Neill et al. 2008; Schmidlen et al. 2019; Solomon et al. 2020; Suckiel et al. 2021; Vogel et al. 2019; Wang et al. 2021; Williams et al. 2018). Studies reported on direct impacts, evidenced by uptake of screening attendance and healthy behaviours (Bowen et al. 2011; Conijn et al. 2020; Hardy et al. 2018; Vogel et al. 2019), positive experiences of genetics services (Brown-Johnson et al. 2021; Christian et al. 2022; Suckiel et al. 2021; Williams et al. 2018) and positive attitudes toward genetics services (Conijn et al. 2020). For example, utilisation of cancer genetic counselling services (Vogel et al. 2019) and mammography screening (Bowen et al. 2011) improved, along with a 22% increase in breast self-exam behaviours (40% before the intervention and 62% after) (Bowen et al. 2011). Positive experiences of genetics services were reported by Brown-Johnson et al. (2021) whereby patients reported an enhanced sense of partnership and accountability with the healthcare team due to digital health. In another study, 88% of 82 participants agreed that the live and interactive webinar would be an acceptable replacement for a one-to-one appointment with a genetics counsellor (Christian et al. 2022). Conijn et al. (2020) also reported on positive attitudes, finding that participants who watched the educational video on expanded carrier screening had a more positive attitude toward preconception screening than those who read only the text description.

Across the literature, expected changes to service engagement could be characterised by attitudes toward or planning to engage (or not engage) with services (Beaudoin et al. 2011; Biesecker et al. 2018; Cragun et al. 2020; O'Neill et al. 2008) or expected changes to the delivery of care (Bangash et al. 2022; Schmidlen et al. 2019; Solomon et al. 2020). For example, in a study with 53 participants, 30.2% had discussed or were planning to discuss the health information from the Genetics Home Reference online resource with physicians (Beaudoin et al. 2011). 28.3% also reported that the information influenced or might influence their future health decisions, and 11.3% either contacted or planned to contact a local support group.

Some negative findings were also reported, whereby participants in the study by Solomon et al. (2020) wanted to be reassured that the interactive features within the digital health tool meant that they would be responded to by the healthcare team and not “lost in cyberspace someplace.” Additionally, parents in the study by Biesecker et al. (2018) who were educated with the web-based platform had stronger decisional conflict (uncertainty) about genetics testing compared to non-parents. Lastly, participants had no significant change in attitudes toward hereditary cancer genetic testing after using a web-based educational tool (Cragun et al. 2020).

#### Empowerment

Only two studies (8%) reported on empowerment (Adam et al. 2018; Cragun et al. 2020), defined by the extent to which an individual believes they have decisional control, cognitive control, behavioural control, hope and emotional regulation (McAllister et al. 2011). The interventions in both studies were for pre-test genetics counselling and used self-reported and subjective empowerment measures. Adam et al. (2018) used the validated and sensitive 24-item Genetic Counselling Outcome Scale (GCOS-24) scale, which measures patient-reported empowerment in clinical genetics services (McAllister et al. 2011). In contrast, Cragun et al. (2020) measured empowerment by asking participants about the extent to which they feel they can make an informed/empowered choice about genetic testing. Both studies reported improvements in empowerment. However, Adam et al. (2018) reported that the DECIDE online decision and educational tool only increased empowerment by less than 2%. Cragun et al. (2020) reported on greater impacts, whereby 74% (227 of 305) of participants felt empowered to make a decision after viewing the web-based educational tool, compared to only 29% before. This finding was statistically significant.

## Discussion

This systematic review synthesised evidence from 24 studies on patient-facing digital interventions for genomics/genetics education, empowerment, and service facilitation. There were promising results regarding educating patients and their families and facilitating engagement with genetics services. However, little literature explored empowerment, with only one study using a validated measure (Adam et al. 2018).

The review also explored for whom and for which purposes the interventions had been developed, finding that the majority of the interventions were delivered in the USA and within clinical environments, while few engaged underserved populations. In fact, only one study focussed on racial/ethnic minorities (Wang et al. 2021), ASL speakers in the deaf community (Boudreault et al. 2017) and students within a Jewish community (Hardy et al. 2018), respectively. This gap in research highlights a need for further engagement with underserved communities that may be at higher risk of genetic disorders or consanguinity. Although rates of consanguinity are decreasing, an estimated 8.5% of children worldwide have consanguineous parents (Shawky et al. 2013) and the practice is still prevalent in parts of the Middle-East, North Africa, and West Asia (Acharya & Sahoo 2021; Bittles 2001; Heidari et al. 2014). In the UK, it was estimated that 59.3% of Pakistani women were married to first or second cousins (Bhopal et al. 2014). However, none of the studies were conducted in these countries or specifically targeted genetic screening or counselling in consanguineous couples.

Additionally, patients often needed to be referred to the intervention by a clinician (e.g., genetics counsellor). This may result in inequitable access, given that there are many barriers to engagement with genetic healthcare services, including a lack of knowledge of genetic services or personal risk (Delikurt et al. 2015; Geer et al. 2001; Willis et al. 2017); education levels (Willis et al. 2017); cost and logistics (Geer et al. 2001; Willis et al. 2017); lack of decision support (Willis et al. 2017) and language and a lack of bilingual providers (Augusto et al. 2019; Kinney et al. 2010). Some groups may also experience stigmatisation or discrimination due to their genetic disorders (Williams et al. 2010). A combination of these factors and potential stigmatisation might create barriers to engagement with genetics services, resulting in groups of individuals who remain underserved and could have been overlooked in the research. This highlights an opportunity for more community-based work and targeted engagement to support equitable access by underserved communities.

Many considerations for technology development were also highlighted within the review. For example, there were only three apps, and more than half of the interventions were web-based, with few having interactive features. It was unsurprising that not many apps had been evaluated, given that our review of 22 patient-facing genetics apps available in the UK identified that none had been formally trialled or tested (Gasteiger et al. 2022). However, it was interesting to note that there was a trend toward more interactive emerging technologies in recent years, with literature published in the last three years reporting on chatbots accessible via smartphones and computers. A key benefit of digital technology is the possibility for interactivity, defined by an element of responsiveness, exchange of information and varying user control (Walther et al. 2005). Interactive features may include personalisation and tailoring, feedback and monitoring, prompts and cues and goal-setting, which are also commonly used behaviour change techniques in digital interventions (Dugas et al. 2020; Taj et al. 2019). Emerging technologies like virtual reality, augmented reality and video games can also foster interactivity through immersion, presence and customisation, while helping make messaging around healthy behaviours more relatable (Hudgens et al.).

Clearly, the literature was dominated by quantitative research (71% of the studies), and although there were six mixed methods studies, there was only one qualitative study. The qualitative work in these studies was important in reporting potential improvements to the content and use of the interventions. For example, some studies reported on language preferences, whereby some participants did not understand the content conveyed (Boudreault et al. 2017), and other users may prefer factual information while some prefer hopeful language (Solomon et al. 2020). The qualitative data also uncovered that some people might want to share the information with family, friends or other patients (Boudreault et al. 2017; Schmidlen et al. 2019; Solomon et al. 2020). This highlights a need for user-friendly language and technical features that enable content to be shared. Further, it supports the need for more qualitative research and co-developed content with participation from future end users to ensure that it meets their expectations and needs, and is inclusive for more under-served communities.

### Recommendations for future research and technology development

This review has highlighted a need for further research that specifically engages underserved groups and communities at-risk of genetic disorders or consanguinity. It is crucial to understand whether and how digital interventions may reach groups who already experience barriers to engaging with genetic services. Additionally, more exploration of patients' experiences with and perceptions of the digital interventions is needed, to understand whether the content is acceptable. Lastly, underlying these research opportunities is a focus on individual and community-based empowerment, which puts patients at the centre of healthcare, and, by definition, means that patients will have increased autonomy over health-related decision-making and actions (Health promotion glossary 1998). Validated patient-reported outcome measures like the GCOS-24 scale which assesses empowerment in genetics counselling and testing services (McAllister et al. 2011) should be employed.

The main implication for the design of future genetics/genomics digital interventions is the inclusion of qualitative co-design or participatory design methods with future end users. Including qualitative methods such as interviews, focus groups, or talk-aloud protocols is vital to understanding user needs, experiences and any usability or acceptability issues early in the design of an intervention. This can allow for an iterative design process during each phase of development and evaluation. Additionally, as explained previously, the incorporation of interactive features is recommended in future interventions through established behaviour change techniques (e.g., tailoring and personalisation, feedback and monitoring, goal setting and prompts/cues). Lastly, content should be simple and not too detailed, addressing a group's average reading age. For example, the average reading age level in the UK is nine years, with 16.4% of adults in England having very poor literacy skills (National Literacy Trust 2023). Producing content at lower reading ages is imperative to ensure it is easy to understand.

Being able to share information with others would also be beneficial. For example, this may include being able to download some of the content (e.g., videos) for private sharing. Other technologies may also link in with email or social media sites, whereby users can directly share content (or the app) with friends and family through their email or sites like Facebook Messenger and Whatsapp.

### Strengths and limitations

A key strength was the inclusion of different study designs, with the results from the mixed methods and qualitative studies providing a richer understanding of patient experiences and perceptions of the digital interventions. The review also builds on previous work, which has typically focussed on one technology (e.g., videoconference in Gorrie et al. (2021)). By taking a broader approach to digital interventions, the review has highlighted how different technologies have been employed and simultaneously identified potential applications for new emerging technologies (e.g., chatbots and precision health) in genetics healthcare. Lastly, the quality appraisals were conducted in a rigorous manner, with almost perfect agreement between the two researchers.

Limitations include that the reviewed papers were limited to the English language. This means that research with underserved ethnic communities might have been missed if it had been published in another language. Additionally, the studies may have been prone to selection and response bias, given that the samples were limited to people who had access to technology and the digital literacy skills to use it. Lastly, digital interventions emerge quickly, so newer interventions may not be represented by the published papers.

## Conclusions

Evidence suggests that digital interventions may be used to deliver information about genetics concepts and conditions, and positively impact service engagement. There is less evidence for empowering patients and engaging underserved communities or consanguineous couples. Future work should focus on co-developing appropriate content with future end users and continue to incorporate interactive features.

## Supplementary Information

Below is the link to the electronic supplementary material.

Supplementary file1 (DOCX 48 KB)
