# Supplementary material for: Digital interventions for genomics and genetics education, empowerment, and service engagement: A systematic review
Source: J Community Genet. 2023 May 18;14(3):227–40. doi: 10.1007/s12687-023-00648-w (PMC10271952; doi:10.1007/s12687-023-00648-w)
Supplement: Supplementary file 1 — Supplementary file1 (DOCX 48 KB) [file 12687_2023_648_MOESM1_ESM.docx]

**Supplementary file**

Table S1. The specific keywords used for the database searches.

| **Population:** Families, carers, patients (non-professionals) | **Exposure:** digital health intervention for genetics education, empowerment, or service engagement | **Outcome:** Understanding, knowledge, awareness, service engagement, empowerment |
| --- | --- | --- |
| Patient*  Famil*  Public  Parent  carer* | Genomic*  Genetic*  Digital health  Web*  App*  mHealth  eHealth  uHealth  Internet  Chatbot  Wearable* | Awareness  Empower*  Educat*  Information  Understand*  Knowledge  Engagement  Use  Access  Uptake |

Table S2. Syntax of three of the database searches.

| **Database** | **Syntax** |
| --- | --- |
| SCOPUS | ( TITLE-ABS ( genomic* OR genetic* ) AND TITLE-ABS ( patient* OR famil* OR public OR parent OR carer* ) AND TITLE-ABS ( digital AND health OR web* OR app* OR mhealth OR ehealth OR uhealth OR internet OR chatbot OR wearable* ) AND TITLE-ABS ( awareness OR empower* OR educat* OR information OR understand* OR knowledge OR engagement OR use OR access OR uptake ) ) AND ( LIMIT-TO ( PUBSTAGE , "final" ) ) AND ( LIMIT-TO ( DOCTYPE , "ar" ) ) AND ( LIMIT-TO ( LANGUAGE , "English" ) ) |
| Embase | (genomic* OR genetic*) AND (patient* OR famil* OR public OR parent OR carer*) AND (digital health OR web* OR app* OR mhealth OR ehealth OR uhealth OR internet OR chatbot OR wearable*) AND (awareness OR empower* OR educat* OR information OR understand* OR knowledge OR engagement OR use OR access OR uptake  limit 1 to (human and english language) |
| PsycINFO | (genomic* OR genetic*) AND (patient* OR famil* OR public OR parent OR carer*) AND (digital health OR web* OR app* OR mhealth OR ehealth OR uhealth OR internet OR chatbot OR wearable*) AND (awareness OR empower* OR educat* OR information OR understand* OR knowledge OR engagement OR use OR access OR uptake) {Including Related Terms} limit 1 to (human and english language) |

Table S3. Characteristics of the included studies.

| **First author; year** | **Setting; country** | **Sample; size; key demographics** | **Study design; data collection methods** | **Key results (education, empowerment, engagement)** |
| --- | --- | --- | --- | --- |
| Adam; 2018 | Tertiary care children’s hospital; Canada | Parents of children with early‐onset (age ≤ 5 years) epilepsy of unknown cause  Sample size: 106   - Adults (30-50 years); Children (0-29 years) - Male (n=15); Female (n=91) - Ethnicity; N/R - 48% (n=37) had at least an undergraduate university degree; n=69 had high school or college certificate | RCT; surveys | - Both DECIDE and conventional genetic counselling significantly increased parents’ knowledge, with no difference between groups. - Empowerment increased but by less than 2% in each group. |
| Bangash; 2022 | Zoom (online); Public access or via Mayo Clinic, USA | Patients (probands with familial hypercholesterolemia diagnosis)    Sample size: 9   - Age range: 30 - ≥ 50; mean: N/R - Male: n=1; 11%; Female: n=8, 89% - Non-Hispanic white: n=9, 100% - Education: High school: n=1, 11%; university: n=8, 89% | Mixed methods; talk aloud (qualitative) and surveys | - 2 of 9 (22%) patients responded that it would significantly improve care - 5 of 9 (56%) responded that it would somewhat improve care - ​​5 of 9 (56%) found information very easy to find, and 7 of 9 (78%) found information very easy to understand. - Wanted more information to be added to the tool. |
| Beaudoin; 2011 | University of Utah Metabolic Service Clinic; USA | Adult patients and parents/guardians of newborns/children with metabolic conditions    Sample size: 53   - Age range: 20–46 (median: 33) - Male: n=14, 26%; Female: n=39, 74% - Non-Hispanic white: n=49, 93%; other (Hispanic, Asian, ‘‘White and Hispanic,’’ and ‘‘Mixed): n=4, 7% - Education: High school graduate or equivalent: n=2, College: n=34, University: n=17 | Quantitative (descriptive); surveys | - Majority (60.4%) reported an improved understanding of an illness or health condition. - 30.2% of participants had discussed or were planning to discuss the health information from the tool with physicians - 28.3% reported that the information influenced or might influence their future health decisions - 11.3% either contacted or planned to contact a local support group. - Wanted more information to be added. |
| Biesecker; 2018 | Clinical (National Institutes of Health); USA | Adults (recruited from the ClinSeq cohort- part of a clinical research cohort for exome sequencing)    Sample size: 459   - Age range: N/A; mean: 63.1 - Hispanic or Latino: 9; Not Hispanic or Latino: 447 (97%) - Other detail: predominantly married, well-educated, post-reproductive, and non-Hispanic white | RCT (1 of 4 arms); surveys | - The web platform was noninferior to the genetic counsellor in terms of knowledge assessed immediately after education and at 1 month and 6 months later. - The web platform was non-inferior to the genetic counsellor on test-specific distress, and decisional conflict about choosing to learn results at 1 and 6 months. - Parents reported statistically significantly greater decisional conflict when educated by the web-based platform (compared to non-parents). - Wanted more information to be added. |
| Boudreault; 2017 | Deaf community, online, conference; USA | ASL speakers in the Deaf Community    Sample size: Focus groups: 43; questionnaires: 97   - Age range: Focus groups: N/R. Questionnaires: mean 44.7 - Gender: Focus groups: N/R. Questionnaires: Males: n=37, 38%, Female: n=60, 62% - Education/ethnicity: Focus groups: N/R. Questionnaires: High school or less: 30, College: 67 - Ancestry: People of colour: 32, Non-Hispanic Caucasian: 63 | Mixed methods; focus groups; questionnaire | - Majority of participants were motivated to become interested in making their own family tree and to tell their family and friends about the video. - Participants did not fully comprehend the material. - Improvements include reducing the content and using simpler language. |
| Bowen; 2011 | Community, online; USA | Community/public sample - women’s breast health    Sample size: 1354   - Age: 43% under 40 years of age - 100% female - 85% Caucasian - 16% only high school educated - Under-sampled those with higher mobility, frequent address changes, and lack of land telephone | RCT; surveys | - Significant improvements in mammography screening compared women in the control group (improvement of 13%). - Women in the intervention group reported statistically significant decreases in interest in genetic testing. - Women in the intervention group reported increased breast self-exams from 40 to 62%. - The intervention effects were more powerful in women who increased breast health knowledge and decreased cancer worry during intervention. |
| Bradbury; 2022 | Community and academic practices; USA | Patients with advanced cancer undergoing tumour only sequencing    Sample size: 472   - Age: Mean: 62.8; Range: N/R - Male: 208 (44.1%); Female: 264 (55.9%) - Hispanic: 6 (1.3%), non-Hispanic: 458 (98.7%), Unknown: 8 - Education: High school or less: 170 (36.0%), University: 302 (64%) - Excluded those without internet access | RCT; surveys | - Patients in the intervention arm had significantly greater increases in knowledge. |
| Brown-Johnson; 2021 | Single academic primary care clinic in community. USA | Patients    Sample size: 50. 16 patients, 9 providers, 2 staff took part in interviews   - Age: Median 47 (24-86) Mean N/R - Female 33 (66%) Male 17 (34%) - Ethnicity - white 25 (50%), Asian 12 (27%), Hispanic 9 (19%), African American 3 (6%). | Mixed methods; chart review; interviews | - Humanwide engaged patients holistically, supported faster medication titration, and strengthened patient-provider relationships (sense of partnership and accountability). - Patients benefited clinically from at least one component e.g., tailored medication from pharmacogenomics; appropriate genetic testing; and/or early identification of risk through remote digital health monitoring. |
| Christian; 2022 | 2 tertiary and 3 outreach sites in Canada | Patients with a clear or suspected clinical diagnosis of hypertrophic cardiomyopathy (HCM)    Sample size: 82   - Age range: 26->65, mean NR - Webinar - 29 (67%) male, 14 (33%) female. Control group 1:1 - 8 (67%) male, 4 (22%) female. Control group in-person - 4 (57%) male, 3 (43%) female - Ethnicity NR. - Education - webinar 7 (16%) high school, college 18 (42%), undergrad 12 (28%), grad 4 (9%), other 2 (5%). 1:1 high school 0, college 5 (42%), undergrad 5 (42%), grad 0, other 2 (17%). In person high school 0, college 1 (14%), undergrad 1 (14%), grad 4 (57%), other 1 (15%) | Non-randomised controlled trial; surveys | - Self-perceived knowledge scores increased by an average of 2.1 for the webinar group and 1.2 for both control groups (in-person group and 1:1 appointment). - Scores for knowledge about HCM, inheritance of HCM and genetic testing for HCM all increased significantly in the webinar group. - 49% (n=21) felt comfortable asking questions during the webinar. - 88% (n=38) indicated that the webinar was an acceptable replacement for a 1:1 appointment |
| Conijn; 2020 | Public, online, Dutch population | Participants in the reproductive age, 18-45    Sample size: 1570   - 789 participants were offered an educational video presenting information on expanded carrier screening (ECS) and mucopolysaccharidosis type III (MPS III). - 781 participants received an educational text related to ECS and MPS III. - A group of 266 participants were additionally recruited in the current study to assess whether attitudes had changed over time due to increased media coverage of ECS - Age: Text: mean 31.2 (7.3) years, range 18-45. Video: mean 31.3 (7.0), range 18-47. - Text: female 379 (48.5%). Video: 411 (52.1%) | Non-randomised, historically controlled study; surveys | - Participants who were offered the video as source of information scored significantly higher on the genetic knowledge test. - Watching the educational video led to an increased intended uptake of expanded carrier screening testing. - Those who watched the video had a more positive attitude toward preconception expanded carrier screening compared to those who read the text. |
| Cragun; 2020 | Vanderbilt Hereditary Cancer Clinic, Nashville | Patients with a personal and/or family history of various cancers are referred by their provider or self-refer to this clinic for cancer genetic risk assessment and GC or testing for any type of cancer-related condition.    Sample size: 305   - Mean 47 years - Female 244/305 (80%) - Non-Hispanic white 257 (86%). High health literacy 231 (76%). Private insurance 232 (76%) | RCT; surveys | - Significant increase in knowledge after viewing the tool. - Significantly increased feelings of empowerment to decide about genetic testing. 29% felt empowered before viewing the tool and 74% felt empowered after. - No significant change in attitudes toward hereditary cancer genetic testing. |
| Gornick; 2018 | 22 surgical practices in 4 states (California, Georgia, Michigan, and Tennessee) | People with early early-stage breast cancer to test for BRCA 1/2  Sample size: 537   - Age: 21-84. Control: 57.03 +/- 10.88. Intervention: 56.52 +/- 10.72 - Gender: not specified - Race: White control 212 (79%) int 210 (79%). Black control 45 (17%) intervention 42 (16%). Other control 13 (5%) intervention 15 (6%). - Education: High school graduate or less control 58 (21%) intervention 57 (21%). Some college/ college graduate control 145 (54%) intervention 148 (55%). Some/completed graduate school control 67 (25%) intervention 62 (23%). - Married/Partnered: No control 83 (31%) intervention 64 (24%), Yes control 187 (69%) intervention 203 (76%) | RCT; surveys | - Significantly more patients in the intervention group compared with the control group had knowledge regarding their BRCA1 and/or BRCA2 probability. - Patients who viewed the intervention had higher odds than those of the control group of correctly answering the question regarding the probability of having a BRCA1 and/or BRCA2 pathogenic variant. |
| Hardy; 2018 | 10 universities across America | Undergraduate Jewish students  Sample size: 1794   - Age: not specified - 'college age' - Approximately 44% were male and 56% were female. - Over 80% reported an Ashkenazi Jewish background (at least 1 Ashkenazi Jewish grandparent). The remainder reported Sephardi Jewish, Mizrahi Jewish, and non-Jewish or mixed ancestry. | Quantitative non-randomized study (pretest posttest); surveys | - Secondary analysis showed a greater change in percent correct overall (from 86.92 ± 14.20 to 90.80 ± 12.40; a difference of 3.9%; p < 0.0001). There were statistically significant improvements in quiz scores for 4 questions (of 7). - Telehealth technology facilitated access to professional genetic counselling services. - Approximately 79% of participants received post-test genetic counselling via phone or video conference. |
| Hernan; 2020 | Division of Clinical Genetics at The Children’s Hospital of NYC | Parents of patients being offered clinical exome sequencing (CES)  Sample size: 207   - Patients mean age 8.4 years. Parents mean age 41 years. - Patients 102/167 male (61%). Parents 132 (79%) female, 32 (20%) male. - 92 (55%) white, non-Hispanic. 73 (44%) all other races. 2 (1%) NR. - Education <college 49 (29%). >college 117 (70%). 1 (1%) NR. - Employed 107 (64%). Unemployed/homemaker/retired/disabled 60 (36%). - Married 126 (75%). Not married 40 (24%). 1 (1%) NR. Depression 21 (13%). Anxiety 20 (12%). | RCT; surveys | - No significant differences between parents in the video and no-video groups on genetics or clinical exome sequencing knowledge. Parents’ scores on genetics knowledge questions were lower in the video than no-video group. |
| Nazareth; 2021 | 180 clinics across USA | Patients scheduled for appointments at 180 clinics across USA  Sample size: 61,070   - Age: ＜18 - 440 (0.7); 18–24 3,476 (5.7); 25–39 13,697 (22.4); 40–60 27,759 (45.5); Older than 60 15,279 (25.0); Unknown 419 (0.7) - Female 58,781 (96.3) Male 1,242 (2.0) Unknown 1,047 (1.7) - Ashkenazi Jewish 269 (0.4), Asian 2,548 (4.2), Black 5,927 (9.7), Hispanic 6,626 (10.9) , Mediterranean 39 (0.06), Native American 87 (0.1) , White 35,910 (58.8), Other 1,085 (1.8), Multiple 4,220 (6.9) , Unknown 4,359 (7.1) | Quantitative descriptive (observational); surveys, usage metrics | - The chatbot helped identify patients at high risk for hereditary cancer syndromes before routine care appointments. - 11,126 users (20.4% of risk assessment completers) met hereditary breast and ovarian cancer criteria, 1,300 (2.4%) users met Lynch criteria, 41 (0.08%) users met polyposis criteria, and 443 (0.8%) reported a hereditary breast and ovarian cancer-, Lynch-, or polyposis syndrome-associated familial variant. - 71.4% of users completed the genetic testing education section and reported high acceptability. |
| O’Neill; 2008 | Thoracic Oncology Clinic at the H. Lee Moffitt Cancer Center and Research Institute, USA | Patients and relative smokers  Sample size: 232 (116 patients, 116 relative-smokers)   - Relative smokers mean age was 38 years (range, 20–54) - 53% female - White (96%), married or living as married (66%), and employed full-time (72%) | Quantitative non-randomised (cohort study); surveys | - 48% (n = 28) made an informed decision, with 21 making an informed decision to be tested and 7 making an informed decision not to be tested. - 52% (n = 28) made less-informed decisions, including participants who were tested and had adequate knowledge, but whose negative attitudes toward testing were not concordant with their decision to be tested (n = 20), followed by those inclined toward testing given adequate knowledge and positive attitudes, but who were not tested (n = 4). - No significant differences in genetics knowledge (about the GSTM1 enzyme and testing for it) between those that did and did not log onto the intervention. |
| Prado; 2018 | Large tertiary care medical centre (Brigham and Women’s Hospital, Boston, Massachusetts) | First degree relatives of patients with rheumatoid arthritis (RA)  Sample size: 238   - Age, mean SD years: Comparison arm (n=80) 43.4 (14.7), PRE-RA 45.0 (14.9), PRE-RA PLUS 48.3 (13.7). - Education > high school: Comparison arm (n=72, 90%), PRE-RA arm (n=68, 87.2%), PRE-RA Plus arm (n= 69, 86.3%). - White - Comparison arm n=69 (86.3%), PRE-RA arm n=65 (83.3%), PRE-RA Plus arm n=73 (91.3%) | RCT; surveys | - Before education, few relatives identified behavioural RA risk factors. - After education, the RA knowledge score increased in all arms, higher in PRE-RA and PRE-RA Plus than comparison groups. - PRE-RA subjects were more likely to identify risk factors than those who received standard education. |
| Schmidlen; 2019 | Central, south central, and north eastern Pennsylvania, USA | Community/public - general genetic health  Sample size: 62   - Age range 18-76+ - Female (*n* = 42, 68%) - Caucasian (*n* = 58, 94%) - College-educated (*n* = 33, 53%), retirees (*n* = 38, 61%), age 56 years or older (*n* = 52, 84%) | Mixed methods; focus groups, survey | - Qualitative analysis revealed four main themes: overall impressions, suggested improvements, concerns and limitations, and implementation. - Participants were favourable to the use of the cascade chatbot and several wanted to utilise the tool to share genomic test results with select family members. Comments alluded to how the chatbot knows ‘all of the relevant clinical details regarding the results’ and can answer relative's questions. - Participants supported using chatbots to consent for genomics research and to interact with healthcare providers for care coordination following genomic results. - Wanted more information to be added. |
| Solomon; 2020 | City of Hope National Medical Center; USA | Patients with cancer  Sample size: 13 (8 patients, 5 family members)   - Median age 55 (range: 38-74) - Female: 6 (75%), Male: 2 (25%) - Education: ≤Middle school: 1, ≤High school: 2, Some college: 3, Unknown: 2 | Mixed methods; focus groups, survey | - Patient/family participants were enthusiastic about the tool. They believed the tool would help them better understand their cancer and treatment. - Patients thought the tool might be helpful for sharing information with their family, friends, and other patients with cancer. - All participants said that they would like to access the tool at home to review it after a clinic visit and refer to it over time. - Participants wanted reassurance that their contact would be responded to. - Mixed reactions about prognostic information (factual vs. hopeful). |
| Suckiel; 2021 | Clinical settings; USA | Paediatric neurologic, immunologic, or cardiac disorders  Sample size: 18   - Age: Mean: 44; range 28–56 years - African American: 2 (11%), Hispanic/Latinx: 10 (56%), European American: 5 (28%), More than one ancestry: 1 (6%) - Education: Less than high school graduate: 5 (28), High school graduate: 6 (33), Vocational program; 1 (6), associate degree: 3 (17), Bachelor’s degree: 2 (11), Doctoral degree: 1 (6) - Annual household income: <$39,000: 8 (44), $40,000–$79,000: 2 (11), $80,000+ : 5 (28), Preferred not to answer: 3 (17) | Qualitative; interviews | - Parent-participants felt that GUÍA made receiving information about the result manageable and appreciated reading along as the counsellor discussed the results. - All parent-participants (N=10) with experience with genetic testing agreed that using GUÍA improved result disclosure. - 17 (94%) said the content was clear and everyone stated that the amount of information was right. |
| Vogel; 2019; | Gynecologic Cancer Clinic; USA | Women (untested with a history of epithelial ovarian, primary peritoneal or fallopian tube cancer)  Sample size: 104   - Age: Mean: 60.9 (intervention group); 62 (control group) - White or non-Hispanic: 93 (89%) - Other: 11 (11%) - Education: No high school: 1 (1%), High school and above: 103 (99%) - Participants were primarily white, non-Hispanic, older, and had at least some college education. | RCT; surveys | - Compared to controls, women randomised to the mAGIC intervention demonstrated greater knowledge of hereditary cancer, which persisted for at least 3 months. - Utilisation of cancer genetic counselling services improved in both study arms, but there was no statistically significant difference. - Participants in the intervention group reported statistically significantly greater confidence in making an appointment for genetic counselling. |
| Wang; 2021 | Boston Medical Center; USA | Patients - vulnerable patient population  Sample size: 273   - Female: 183 (67.0%); Male: 90 (33.0%) - Hispanic/Latino: 48 (17.6%), Caucasian: 39 (14.3%), African American: 154 (56.6%), Asian: 6 (2.2%), Other/multiple: 21 (7.7%) - Education: No high school: 50 (18.3%), High school graduate/GED:79 (28.9 %), Some college: 62 (22.7%), College degree: 54 (19.8%), Graduate or doctorate: 28 (10.3%) - Literacy: High likelihood of limited literacy: (35.2%) Possible limited literacy: (38.8%) - ≤$25,000: 141 (52.8%) | RCT; usage metrics, concordance between tool and genetic counsellor | Concordance (compared to a genetic counsellor) was significantly greater for participants randomised to VICKY (virtual counsellor) compared to the control arm for ascertaining first and second-degree relatives. |
| Wierstra; 2018 | Community, university and IBD clinic; USA | Patients and people (18-45 years) attending an IBD clinic  Sample size: 78   - Age range 25.6–32.9 - Male: 15 (19.2%), Female: n = 63 (80.3%) - Ethnicity: N/R - English as first language: 71 (91%) - Education: Grade 12: 9 (11.5%), Some postsecondary: 15 (19.2%), Bachelor’s degree: 28 (35.9%), Graduate degree: 14 (17.9%), Technical/trade school degree: 12 (15.4%) - Income: Less than $20, 000: 8 (10.4%) $20,000 to $39,999: 3 (3.9%) $40,000 to $69,999: 23 (29.9%) $70,000 to $99,999: 41 (53.2%) $100,000 or more: 2 (2.6%) | RCT; surveys | - The online educational portal significantly improved and maintained IBD-specific reproductive patient knowledge for more than 6 months. |
| Williams; 2018 | Genetic counselling service; USA | Parents of children with undiagnosed Intellectual Disability, Autism Spectrum Disorder and/or multiple congenital anomalies  Sample size: 52   - Male: 21 (40.3%), Female: 29 (55.9%), Missing: 2 (3.8%) - White or Caucasian: 49 (94.3%), Missing: 1 (1.9%), Hispanic: 2 (3.8%) - Education: Some high school (9–12:): 4 (7.7%), High school graduate or GED: 10 (19.2%), Post high school training other than college: 7 (13.5%), Some college: 13 (25.0%), Bachelor’s degree or equivalent: 9 (17.3%), Master’s degree: 8 (15.4%), Doctor or other professional degree: 1 (1.9%) - Less than $15,000: 1 (1.9%), $15,000 to $29,999: 4 (7.7%), $30,000 to $44,999: 6 (11.5%), $45,000 to $59,999: 8 (15.4%), $60,000 to $89,999: 18 (34.6%), $90,000 to $149,999: 5 (9.6%), $150,000 to $199,999: 5 (9.6%), $200,000 or above: 3 (5.8%), Missing: 2 (3.8%) - Slightly more mothers than fathers completed the baseline survey (58% vs 42%) and most participants were White (96%), married (88%), had at least some college (63%), and were employed (75%). Health literacy and numeracy was high; greater than 60% of parents indicated they were often or always confident filling out medical forms by themselves and greater than 70% indicated they found statistics easy or very easy to understand. | Mixed methods; interviews; surveys | - Some parents reported the value and impact the patient-centred enhanced genomic report had for them and how it improved the interpretation of their child’s complex results. - Customizable template reports may provide a useful and durable source of information that can support and enhance the information provided by genetics professionals in traditional face-to-face encounters. - The parents with children who received a diagnosis, found the enhanced genomic report aided in communication about the genetic finding and the rare condition. |

Table S4. Summary of the digital health interventions reported in the included studies.

| **First author; date** | **Intervention (technology; description)** |
| --- | --- |
| Adam; 2018 | Web-based tool (DECIDE).   - Interactive online educational tool and decision aid to guide adult patients or parents of affected children towards a decision about whether or not to have diagnostic genome‐wide sequencing. Designed for pre-text decision-making. - Provides genomic testing information via a choice of media (text or video), and different levels of detail and takes 10-45 mins to complete. It presents the pros/cons of sequencing, including incidental findings and variants of unknown significance. It guides users to make a logical, but over‐rideable decision. |
| Bangash; 2022 | Web-based tool.   - Interactive website 'FH Family Share' intended to facilitate communication between familial hypercholesterolemia probands (first person to get genetic testing) and their family members. |
| Beaudoin; 2011 | Online resource (Genetics Home Reference- GHR).   - Information Prescription, whereby the resource is prescribed by a physician. - Provides consumers and providers with easy-to-understand, accurate, and reliable health information about genetic conditions. - The website provides information about more than 550 health conditions, diseases, and syndromes. |
| Biesecker; 2018 | Web-based platform.   - Educational content covered what it means to be a carrier, autosomal-recessive inheritance, carrier status for children and grandchildren, the participant’s personal carrier results report, and testing limitations. - Concepts were illustrated using visual aids and the individualised carrier results report included information text bubbles that defined the headings for the results. - Genetic testing reports included genes associated with disorders inherited in an autosomal-recessive pattern. |
| Boudreault; 2017 | Cancer Genetics Education Module video.   - The Deaf Genetics Project team used a bilingual approach to develop a 37-min interactive module video in American Sign Language with closed captions and quizzes. - The content modules were: 1) Introduction: explains that although the focus is on hereditary breast, ovarian, uterine, and colon cancers, the information is applicable to other hereditary diseases. 2) Creating a Family Tree: explains how to develop a pedigree. 3): Risk Factors for Inherited Cancer: covers what a high-risk family tree for cancer might look like. 4): How Cancer Is Inherited. |
| Bowen; 2011 | Website.   - Provides tailored and personalised risk information, followed by offers of additional support if needed. - Participants log into their profiles to access information and facts about breast cancer. They could see messages from the health counsellor and ask them questions. - Mixed and genetic risk women were prompted to sign up for a counselling session to review their risk page. They could also communicate with others and make breast health commitments based on information they provided. For example, a participant who reported never having a mammogram could commit to scheduling one in the next few months. - Women at elevated risk due to family history of breast cancer were invited to participate in introductory genetic counselling (4 sessions). |
| Bradbury; 2022 | Web-based tool (Communication and Education in Tumour Profiling- COMET).   - A theoretically informed, user-tested, self-directed, mobile-ready genetic education intervention. - It has 6 modules consisting of written content and videos that cover genetic information like what DNA is, how testing is beneficial, how testing might impact family/healthcare, risks and benefits of testing and types of genetic test results. |
| Brown-Johnson; 2021 | Multi-modal precision health platform   - Precision health platform using data-driven biomedicine to predict and prevent disease. - Four components: 1) Health coaching 2) Digital health- four wireless tools that connect patient values taken at home to the clinic electronic health record (EHR) for blood-pressure (cuff), weight (digital scale), glucose (glucometer), and activity (smartphone pedometer); 3) Pharmacogenomics using saliva-based screening for drug-gene interactions; and 4) Genetic screening to assess risk of Centers for Disease Control (CDC) Tier 1 markers of hereditary breast and ovarian cancer syndrome, Lynch syndrome, or familial hypercholesterolemia. |
| Christian; 2022 | Webinar.   - A live, patient-facing, interactive, provincial webinar for pre-test genetic counselling for patients with a clear or suspected clinical diagnosis of hypertrophic cardiomyopathy. |
| Conijn; 2020 | Educational video.   - Video for people in reproductive age (18-45) on preconception expanded carrier screening. This aims to identify couples with an increased risk of having a child with an autosomal recessive disorder before pregnancy, thereby enabling reproductive choices. |
| Cragun; 2020 | Web-based tool.   - Educational tool with standard pretest genetic counselling elements related to panel-based testing for multiple genes associated with cancer risk. |
| Gornick; 2018 | Decision tool (iCan-Decide).   - A tailored, comprehensive, and interactive decision tool for people with early stage breast cancer to test for BRCA1/2 |
| Hardy; 2018 | Video and website (JScreen).   - JScreen provides pre-test education via an engaging, animated 3-min video that explains important concepts such as recessive inheritance, availability of reproductive options for at-risk individuals and couples, and limitations of carrier testing. - The JScreen website describes certain diseases on the screening panel that are more common in the Jewish population. |
| Hernan; 2020 | Video.   - Parents of patients offered clinical exome sequencing were offered a pre-session, self-guided video educational tool. |
| Nazareth; 2021 | Chatbot (Genetic Information Assistant- GIA)   - Web-based chatbot used to assess hereditary cancer risk before routine visits with obstetrics and gynaecology, primary care, or routine cancer screening clinics. |
| O’Neill; 2008 | Web-based tool.   - For genetic susceptibility testing for patients with stage IIIB/IV lung cancer and their adult blood relatives aged 18 to 55 who were smokers. |
| Prado; 2018 | Web-based tool (Personalized Risk Estimator for RA (PRE-RA)).   - The tool displayed personalised rheumatoid arthritis risk results (genetics, autoantibodies, demographics, and behaviours) and educated about risk factors. |
| Schmidlen; 2019 | Chatbots (consent chatbot, follow-up chatbot, cascade chatbot)   - Chatbot developed by Geisinger and Clear Genetics, Inc. to facilitate communication with participants receiving clinically actionable genetic variants from the MyCode® Community Health Initiative (MyCode®). - The 3 chatbots included a consent chatbot, patient follow-up chatbot and a cascade chatbot, whereby users can share genetic test results with others. |
| Solomon; 2020 | Web-based tool.   - Patient-directed, genomic sequencing education and return of results tool. - Somatic and germline sequencing results stored in the platform, with embedded multimedia genomic education with interactive features. - Promotes patient and caregiver engagement with genomic information and precision cancer care. - Patient facing genomic report to optimise patient knowledge and improve outcomes. |
| Suckiel; 2021 | Application (Genomic Understanding, Information and Awareness- GUÍA).   - Digital platform designed to facilitate the delivery of GS results and related clinical information to participants and families of diverse backgrounds. - GUÍA allows genome sequencers to walk patients through their genomic test results in a personalised, highly visual, and narrative manner. GUÍA was developed based on the perspectives and input of providers, patients, and community stakeholders as part of the NYCKidSeqProject |
| Vogel; 2019 | Mobile application (mobile Application for Genetic Information on Cancer- mAGIC).   - Provides education and increases utilisation of genetic counselling services among women with ovarian cancer. - Written and video messages focus on increasing participants' self-efficacy by emphasising their ability to change a situation, e.g., seek genetic counselling or talk with family, and effectively manage their emotional reactions. - To encourage buy-in, messages were personalised using a participant's name and when possible, tailored to a specific participant's answers to questions embedded in each lesson. The intervention lasted 7 days and lasted 70-90 mins. |
| Wang; 2021 | Conversational agent (Virtual Counsellor for Knowing Your Family History- VICKY); web-based tool (My Family Health Portrait- MFHP)   - Animated computer character designed to collect a detailed family health history by asking the user a series of questions. - Users respond to VICKY’s verbal questions by selecting from pre-formulated simple responses on a touchscreen, simulating face-to-face conversations between a patient and health care provider. - VICKY 2.0 included a more comprehensive list of health conditions (e.g., heart disease, stroke, cancer, diabetes, hypertension, high cholesterol, mental health issues, schizophrenia, addiction, and alcoholism) - MFHP is widely used for collecting family health histories and uses a traditional interface wherein pop-up windows and drop-down menus are used to facilitate data entry. Conditions listed include heart disease, stroke, cancer, diabetes, hypertension, high cholesterol, and psychological disorders. Other conditions can be added under the “other - add new” option. - Both are available in English and Spanish. |
| Wierstra; 2018 | Website.   - Covers genetics, fertility, surgery, pregnancy, medications, delivery, and postpartum in the context of irritable bowel disease (IBD). - The intervention was 60-day access to the educational portal, which consisted of 5 modules: “I have IBD, can I get pregnant?”; “I have IBD, will my child have IBD?”; “I have IBD, could surgery affect my ability to become pregnant?”; “How does IBD affect pregnancy?”; and “How does IBD affect delivery, postpartum, and breastfeeding?” - Each module consisted of text with references, short (<5 minutes) video clips explaining the topic, a slide deck, and mini-quizzes. |
| Williams; 2018 | Application (GenomeCOMPASS report)   - Software app that enables the presentation of the enhanced genomic report through an interface accessible in the electronic health record (EHR). - Providers view the report while the patient is present. Patients can access the report via the patient portal associated with the EHR. |
